# Repeat-swap homology modeling of secondary active transporters: updated protocol and prediction of elevator-type mechanisms

**DOI:** 10.3389/fphar.2015.00183

**Published:** 2015-09-04

**Authors:** Ariela Vergara-Jaque, Cristina Fenollar-Ferrer, Desirée Kaufmann, Lucy R. Forrest

**Affiliations:** Computational Structural Biology Section, Porter Neuroscience Research Center, National Institute of Neurological Disorders and Stroke – National Institutes of Health, Bethesda, MDUSA

**Keywords:** neurotransmitter, membrane protein, secondary transport, alternating access, asymmetry exchange, glutamate, concentrative nucleoside transporter

## Abstract

Secondary active transporters are critical for neurotransmitter clearance and recycling during synaptic transmission and uptake of nutrients. These proteins mediate the movement of solutes against their concentration gradients, by using the energy released in the movement of ions down pre-existing concentration gradients. To achieve this, transporters conform to the so-called alternating-access hypothesis, whereby the protein adopts at least two conformations in which the substrate binding sites are exposed to one or other side of the membrane, but not both simultaneously. Structures of a bacterial homolog of neuronal glutamate transporters, Glt_Ph_, in several different conformational states have revealed that the protein structure is asymmetric in the outward- and inward-open states, and that the conformational change connecting them involves a elevator-like movement of a substrate binding domain across the membrane. The structural asymmetry is created by inverted-topology repeats, i.e., structural repeats with similar overall folds whose transmembrane topologies are related to each other by two-fold pseudo-symmetry around an axis parallel to the membrane plane. Inverted repeats have been found in around three-quarters of secondary transporter folds. Moreover, the (a)symmetry of these systems has been successfully used as a bioinformatic tool, called “repeat-swap modeling” to predict structural models of a transporter in one conformation using the known structure of the transporter in the complementary conformation as a template. Here, we describe an updated repeat-swap homology modeling protocol, and calibrate the accuracy of the method using Glt_Ph_, for which both inward- and outward-facing conformations are known. We then apply this repeat-swap homology modeling procedure to a concentrative nucleoside transporter, VcCNT, which has a three-dimensional arrangement related to that of Glt_Ph_. The repeat-swapped model of VcCNT predicts that nucleoside transport also occurs via an elevator-like mechanism.

## Introduction

Neurotransmitters are required for signal transduction between neurons; however, to prevent continuous signaling and neuronal toxicity, they are rapidly removed from the synaptic cleft by membrane transporters. Transporter proteins are also required for TM movement of essential nutrients such as citrate, into the brain. The involvement of transporters in diseases such as depression and autism, render them important therapeutic targets for neurological and psychiatric disorders. Secondary transporter proteins mediate the movement of neurotransmitters and nutrients against their concentration gradients by using the energy released in the movement of ions down pre-existing concentration gradients. To achieve this, transporters conform to the so-called alternating-access hypothesis, whereby the protein adopts at least two conformations in which the substrate binding sites are exposed to one or other side of the membrane, but not both simultaneously.

A significant bottleneck in the design of therapeutic treatments, however, is the limited amount of structural information available for these transporters. The overexpression and crystallization of membrane proteins is notoriously challenging (Bolla et al., 2012). Transporter proteins in particular, tend to crystallize in only one of the two major states, making it difficult to resolve their structures in the other conformations, and limiting our understanding of transporter mechanism. For example, the structure of a sodium-coupled dicarboxylate transporter from *Vibrio cholerae*, VcINDY, which is related to the neuronal citrate transporter NaCT, has only been determined in one conformation to date (Mancusso et al., 2012). In this regard, Glt_Ph_, a homolog of the neuronal EAATs of the SLC1 family, from *Pyrococcus horikoshii*, has become an important model system. The relatively large number of X-ray structures of Glt_Ph_ in different states (Yernool et al., 2004; Reyes et al., 2009; Verdon and Boudker, 2012; Verdon et al., 2014) makes this transporter an ideal system for the study of transport mechanism in neuronal transporters, from a structural point of view.

Analysis of the structure of Glt_Ph_ revealed an inverted-repeat architecture (Yernool et al., 2004; Crisman et al., 2009; Boudker and Verdon, 2010), a feature observed in around three-quarters of secondary active transporters of known fold (see Forrest et al., 2011; Forrest, 2015 for reviews). Specifically, large segments of the polypeptide chain are related by structure, but are oriented with the opposite TM topology. This confers the structure with an inherent two-fold pseudo-symmetry, which turns out to be related to the ability of the transporter to adopt two major conformations during the transport cycle, namely outward- and inward-facing states. Specifically, the two repeats adopt a distinctive asymmetry in their conformations, which results in the overall formation of, e.g., an outward-facing state (Fleishman et al., 2006; Chen et al., 2007; Forrest et al., 2008; Crisman et al., 2009; Radestock and Forrest, 2011; Liao et al., 2012). By exchanging their conformations, the two repeats can create a new asymmetric state that is open to the opposite side of the membrane, e.g., inward-facing (Fleishman et al., 2006; Chen et al., 2007; Forrest et al., 2008; Crisman et al., 2009; Radestock and Forrest, 2011; Liao et al., 2012; Schushan et al., 2012). We refer to this process of alternating access by inverted-topology transporters as “asymmetry exchange.”

The asymmetry-exchange concept has been successfully used as a bioinformatics tool to obtain models of a given conformation using the X-ray structure of the transporter in the complementary conformation as a template (Forrest et al., 2008; Crisman et al., 2009; Radestock and Forrest, 2011; Liao et al., 2012; Schushan et al., 2012; Yaffe et al., 2013; Fowler et al., 2015). In this so-called repeat-swap homology modeling technique, the first structural repeat is modeled using the second structural repeat as a template, while the second repeat is concurrently modeled using the first repeat as a template. Given the difficulties of transporter crystallization mentioned above, the ability to predict one state of a transporter once structural data is available for the complementary state provides a very valuable tool.

This repeat-swap technique was first applied to the bacterial amino-acid transporter LeuT from *Aquifex aeolicus*, which is a homolog of neuronal NSSs from the SLC6 family, such as SERT. Comparison of the outward-facing X-ray structure with the inward-facing model of LeuT accurately predicted the cytoplasmic pathway in NSS transporters (Forrest et al., 2008), and led to the proposed “rocking-bundle” mechanism (Forrest and Rudnick, 2009). This mechanism involves the movement of a so-called “transport” domain (which in the case of LeuT, consists of a four-helix bundle, leading to the name “rocking bundle”), relative to a “scaffold” domain, which remains essentially static with respect to the membrane. Although the rocking-bundle concept is somewhat simplistic, the major states of the transport cycle in LeuT-like transporters were subsequently found to be consistent with the overall changes predicted by the repeat-swap mechanism, at least with respective to the relative positions of domains (Weyand et al., 2008; Shimamura et al., 2010; Krishnamurthy and Gouaux, 2012). Similar rigid-body movements, also referred to as a “rocker-switch” mechanism, have been proposed for other transporter families such as the major facilitator superfamily (Abramson et al., 2003; Radestock and Forrest, 2011). In all these cases, structures of the end states confirm that the asymmetry exchange hypothesis predicts the changes in relative positions of domains (see e.g., Dang et al., 2010; Radestock and Forrest, 2011), and predicts the helices that line the access pathways.

One of the most dramatic conformational changes identified in a secondary transporter has been for Glt_Ph_. The mechanism for Glt_Ph_ is unlike the aforementioned mechanisms, in which the substrate is expected to maintain its position, while domains in the protein called “gates” open and close on either side of the substrate binding site. Comparison of outward- and inward-facing structures of Glt_Ph_ instead reveals a mechanism in which a domain containing the substrate binding site (known as the “transport” domain) moves up and down within the protein, reminiscent of an elevator or a piston, in order to transport the substrate and ion binding sites across the hydrophobic barrier provided by the protein and membrane (Reyes et al., 2009). Specifically, the transport domain moves perpendicular to the membrane plane by ~16 Å, and rotates by ~40°. Remarkably, even this extensive conformational change was predicted by a repeat-swap modeling procedure, in which a cytoplasm-facing state of the protein was modeled using the inverted repeats from a Glt_Ph_ crystal structure in an outward-facing conformation as a template (Crisman et al., 2009; Forrest et al., 2011).

The procedure used by Crisman et al. (2009) was developed *ad hoc* since the goal of that study was to provide proof of principle that repeat-swapping could lead to an alternate-conformation model of a transporter with such a complex fold. In the present work, we describe a refined and standardized repeat-swap homology modeling protocol. We then focus on the application of this protocol to proteins that potentially undergo an elevator-like movement during their transport cycle, although the updated protocol is applicable to any protein with an inverted-repeat topology, independent of the type of conformational mechanism. The availability of X-ray structures of Glt_Ph_ in both outward- and inward-open conformations makes this protein a suitable model system on which to validate, refine, and calibrate the protocol. We therefore use the protocol to predict the outward-facing conformation of Glt_Ph_ based on an inward-facing conformation, i.e., the converse of the model built during the previous work (Crisman et al., 2009). The model constructed using the refined protocol differs by ~4 Å (averaged over all Cα positions) from the X-ray structure in the same conformation, which is a remarkably small error given that the conformational changes are on the order of 15–20 Å. We demonstrate that a large proportion of that “error” is in fact a difference in the extent of the excursion, which depends on the initial state that is used as a template.

**FIGURE 1:**
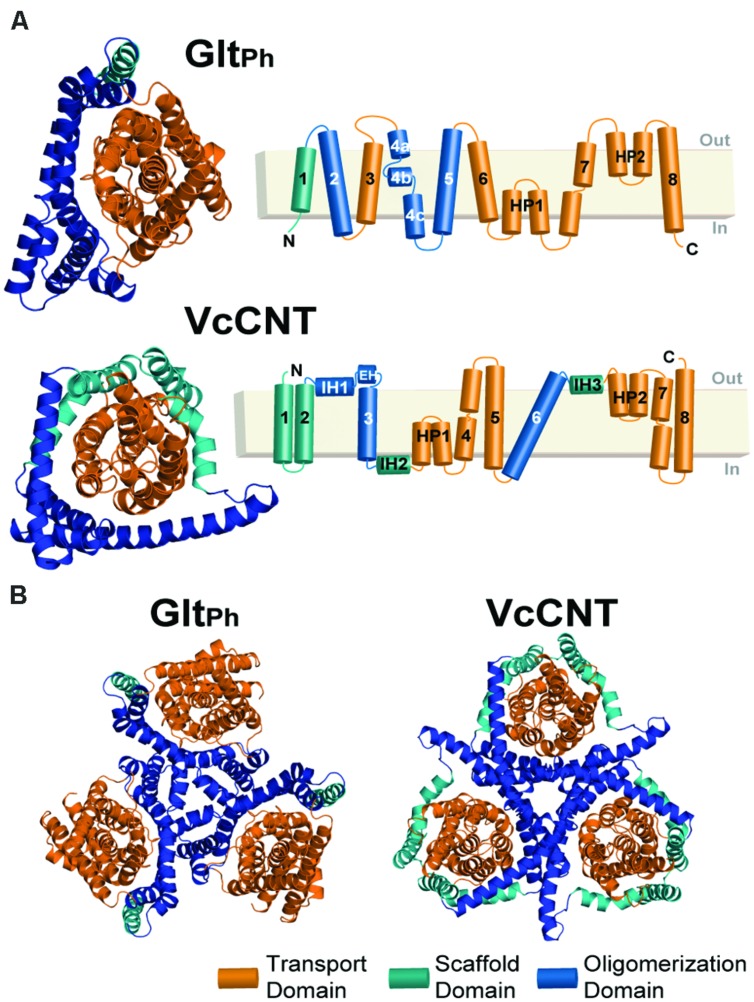
**Structure of the Glt_Ph_ and VcCNT transporters. (A)** Cartoon representation of the Glt_Ph_ (top) and VcCNT (bottom) protomer structures shown from the extracellular side of the membrane (left). HP, helical hairpin; IH, interfacial helix; EH, extracellular helix. In both proteins, the transport domain is colored orange. The scaffold domain is colored cyan and blue, with the blue regions indicating helices involved in oligomerization. The helices forming each domain are detailed in the topology diagrams to the right. **(B)** Trimeric structures of Glt_Ph_ (left) and VcCNT (right) shown from the extracellular side of the membrane.

We then apply the standardized protocol to VcCNT, a CNT from *V. cholerae*, whose structure is available only in an inward-facing conformation (Johnson et al., 2012). VcCNT has also been proposed to transport its substrate and ions via an elevator-like movement. Although not related by sequence, or even TM topology (Johnson et al., 2012), several features of VcCNT are similar to those of Glt_Ph_, namely that it appears to comprise scaffold/oligomerization and transport domains, as well as helical hairpin elements that dip into, but do not cross, the membrane (**Figure 1**). In addition, comparison of the model obtained for VcCNT in an outward-open state to the inward-open X-ray structure, suggests that a similar elevator-like mechanism also occurs in the CNT transporter family. Our results therefore show that the new protocol may be useful for predicting conformational mechanisms in other neuronal transporters whose structures that are undoubtedly to follow. Such information will be important in the design of effective therapies targeted to neuronal transporters.

## Materials and Methods

**FIGURE 2:**
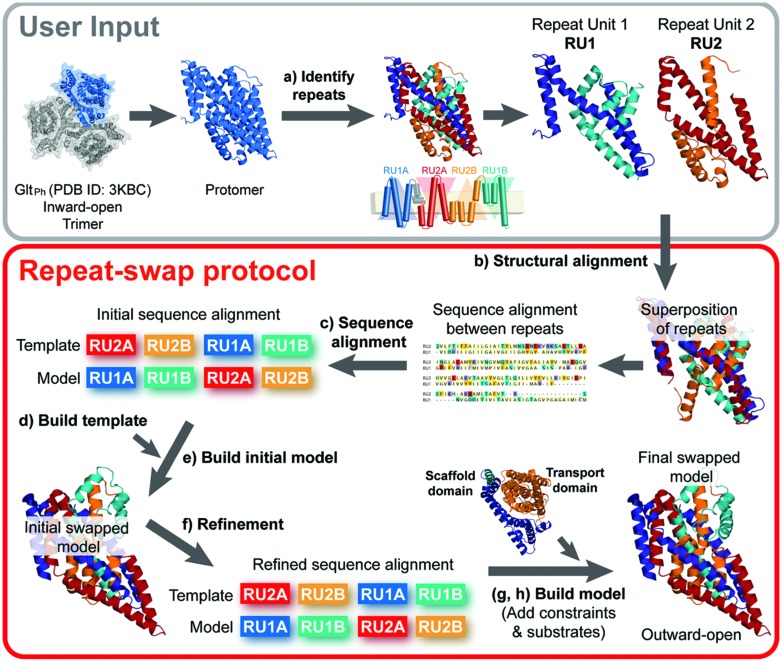
**Repeat-swap homology modeling protocol**. To build a molecular model of membrane transporters with internal structural pseudo-symmetry, the crystal structure of a protomer of the protein (e.g., Glt_Ph_) is required as an input. In step (a) the protomer must be carefully analyzed in order to identify the internal repeats. This information is often provided by the authors that report the structure, however, additional insights may be obtained using SymD and CE-symm. In preparation for the modeling, (b,c) an initial sequence alignment is built from a structural alignment of the repeat units (e.g., RU1 and RU2) using a structure alignment program such as TM-Align. In addition, (d) a template structure is built by changing the order of the repeat units in the PDB file and including the regions of the protein that do not belong to the repeat units. At this point, (e) an initial swapped model of the protein can be built, using Modeler. (f) The input alignment is then carefully refined to optimize the agreement with the secondary structure definition and the evolutionary conservation pattern, obtained with DSSP and ConSurf, respectively. Once a model is built using the optimal alignment, the scaffold and transport domains are identified, where possible. (g) Cα-Cα distance restraints can then be defined for each of these domains according to the crystal structure values, and the model is rebuilt using those restraints. Finally, (h) substrates are added to the binding site, if known, and the final repeat-swapped model can be compared with the crystal structure.

**FIGURE 3:**
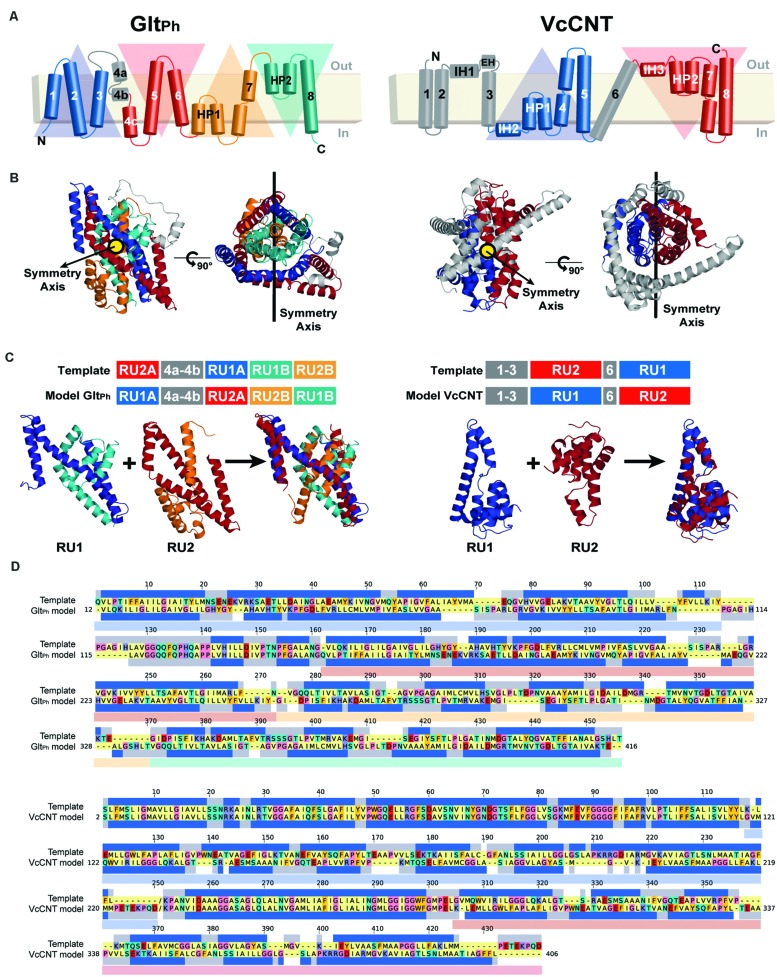
**Repeat-swap modeling of the Glt_Ph_ and VcCNT transporters in alternate conformations. (A)** A schematic representation of the topology of each protein is shown colored according to the structural repeats. In Glt_Ph_, the helices on the blue and cyan triangular backgrounds comprise repeat unit 1 (RU1), while repeat unit 2 (RU2) is composed of the helices on the red and orange triangular backgrounds. In VcCNT, the blue helices form RU1 and the red helices comprise RU2. **(B)** In both cases, RU1 is related to RU2 by two-fold pseudo-symmetry, with the symmetry axis (black line) parallel to the membrane. **(C)** A structural alignment, built with TM-Align, of the repeats of each protein is shown in cartoon representation with the helices colored according to the topology. The initial sequence alignment used to build a swapped-repeat model was generated based on this structural alignment. A schematic of the sequence alignment is shown above, with peripheral helices in the model and template shown in gray. **(D)** Refined sequence alignment between model and template sequences. The percentage of identical sequences is 21.2 and 46.4% for Glt_Ph_ and VcCNT, respectively, for the full length alignment, including peripheral elements; within the repeats, 14.0 and 7.3% of the residues are identical. Note that the template sequence is the input X-ray protein structure with the order of the repeats reversed. The sequence range of each repeat is displayed with transparent rectangles under the target sequences colored according to the triangular backgrounds in **(A)**. The secondary structure (helix) assignment obtained with DSSP for the X-ray structure is indicated by dark blue rectangles.

**FIGURE 4:**
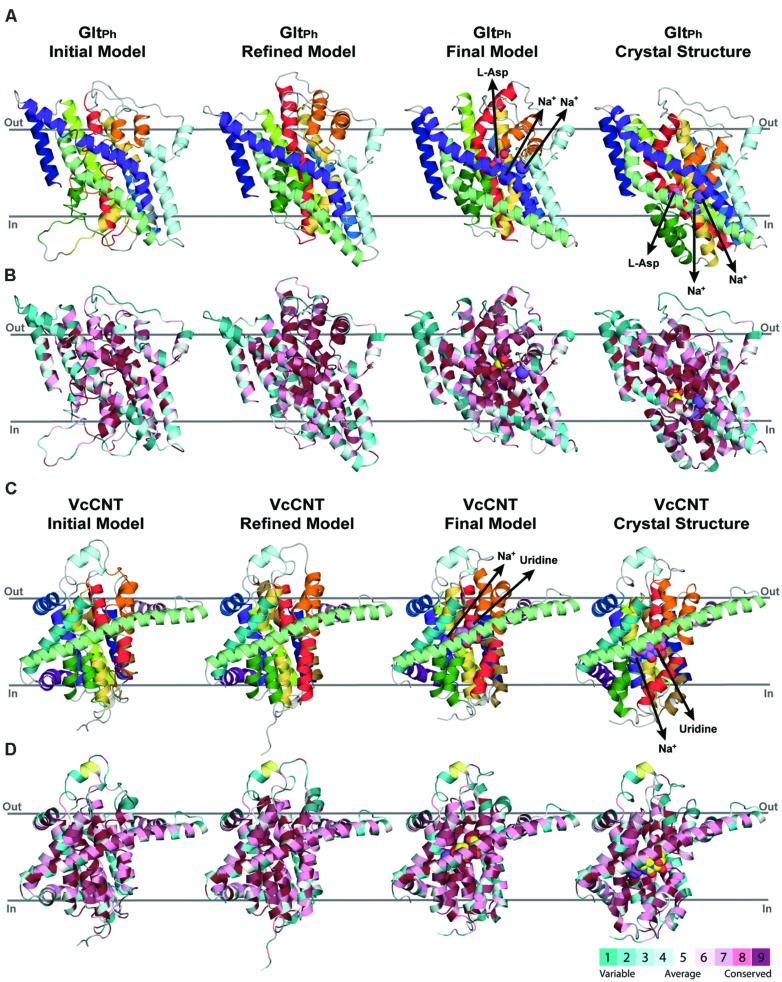
**Refinement of repeat-swapped outward-facing models of Glt_Ph_ and VcCNT compared with input inward-facing structures.** Models of Glt_Ph_ **(A,B)** and VcCNT **(C,D)** built using initial or refined alignments, both before and after applying restraints within the scaffold and transport domains. Models are shown as cartoon helices, colored with separate colors for each helix (see **Figure 5**) in **(A,C)**, and using ConSurf coloring in **(B,D)**. Substrates are shown as spheres, with their pathways illustrated using arrows. ConSurf conservation patterns are mapped to the helices using colors from cyan (most variant residues) to dark pink (most conserved residues); yellow indicates positions with insufficient sequence information to allow a conservation assignment. As seen for the X-ray structures (*right*), conserved residues cluster on the inside of the protein, and at oligomerization interfaces, while variant residues are found primarily on the surface. This observation, and the patterns seen in the template structure were used to guide the refinement of the alignments. The position of the proteins in the membrane was determined with the OPM server.

The eight steps (a–h) of the standardized repeat-swap homology modeling protocol, illustrated in **Figure 2**, are as follows:

(a) *Identification of structural repeat units*. The starting point of this methodology is the identification of the residues that constitute each of the inverted repeats in the known structure (**Figure 3A**). The underlying assumption is that these repeat units adopt different internal and relative conformations in order to create the asymmetry of an outward- or inward-open state; exchanging between these two conformations is therefore what enables the major conformational change required for TM transport. To date, no reproducible strategy has been developed to identify structural repeats reliably, mainly because the asymmetry and low sequence identity between the domains can stymie even the most sensitive structure alignment programs. Thus, the repeats may be identified in a number of, essentially manual, ways. Two tools are available to aid this process: SymD (Tai et al., 2014) and CE-symm (Myers-Turnbull et al., 2014), which take as input a single protein structure file in Protein Data Bank (PDB) format, and then report internal pseudo-symmetry within the structure (**Figure 3A**). Progressively dividing the structure into smaller domains can be a useful strategy for identifying smaller repeated elements such as those in Glt_Ph_ (**Figure 3A**).
(b) *Structural and sequence alignment of the repeat units*. After structural superimposition of the repeat fragments using a structure alignment algorithm (e.g., TM-Align Zhang and Skolnick, 2005), a sequence alignment between the repeats can be extracted, based on the pairs of residues that are close in space in the structure alignment. Such a pairwise sequence alignment is typically reported by the structure alignment program.
(c) *Preparing the initial full-length sequence alignment*. The initial sequence alignment of the full-length protein to the template is constructed by duplicating the sequence alignment obtained in step b. Specifically, the first repeat of the protein is modeled based on – and is therefore aligned to – the second repeat, while the second repeat is modeled based on the first repeat (see **Figure 3C**).
(d) *Preparing the template coordinate file*. The “swapped” template used in this procedure is obtained by simply changing the order of the repeats in the PDB-formatted coordinate file: in this way, the first part of the coordinate file contains the residues from the second repeat, and is followed by those that constitute the first repeat. To model fragments of the protein that are not part of the structural repeats and therefore have no counterpart (e.g., helices 4a and 4b in Glt_Ph_, **Figures 3A,B**, or other peripheral TM helices), the template can be the same fragment in the X-ray structure (**Figure 3C**). Note that a characteristic of the repeat-swap modeling process is that the model obtained is oriented “upside-down” with respect to the template (for example, in Glt_Ph_, the N-terminal end of TM1 is modeled to have the same coordinate space as the N-terminal end of TM4c in the template); as a consequence an additional step is required to reorient the additional or peripheral template fragments to the position in space that they need to be in the model. In practice, structurally aligning the X-ray structure onto an initial swapped model using the same repeats (e.g., repeat 1 in the X-ray onto repeat 1 in the model) is a straightforward way to reorient the peripheral segments; these must then be added to the template coordinate file in the new orientation, for inclusion in a subsequent round of modeling. The approximate orientation of these peripheral segments will be corrected by adding restraints in a later step of the protocol (step g). Once all the pieces are assembled in order, the residues in the new “swapped” template coordinate file need to be renumbered.
(e) *Constructing an initial structural model*. The initial full-length alignment, with the sequence of the additional or peripheral fragments correctly included, is used as a guide to build a preliminary homology model based on the “swapped” template.
(f) *Refinement of the sequence alignment*. The initial sequence alignment typically requires refinement, due to the sequence and structural divergence of the repeats, which can make them difficult to align. A common step is to remove gaps within secondary structural elements, where the secondary structure is assigned according to DSSP (Kabsch and Sander, 1983) for the known structure (**Figure 3D**). In addition, the conservation patterns obtained from the ConSurf web server (Ashkenazy et al., 2010) can be mapped onto the initial model (**Figures 4B,D**), and used to position the most conserved residues so that they are packed inside the protein, while sequence-variant positions face the exterior, mimicking the arrangement in the input X-ray structure. The model built with the refined alignment is useful for identifying the scaffold and transport domains in the case of a two-domain elevator-like mechanism; superimposing the preliminary model onto the known structure using only helices that form the oligomerization interface (**Figure 1**) can provide a first indication of the mobile elements of the protein.
(g) *Refinement of the model by introducing distance restraints*. Additional distance restraints between Cα atoms taken from the known structure can be used in the repeat-swap modeling process. These restraints are intended to conserve the native helical arrangement and intramolecular packing within domains that move relative to one another, reducing the deviations that arise due to the sequence divergence of the repeats. They also help to fix the orientation of any peripheral segments (see blue helices in **Figure 4C**). However, identification of suitable restraints requires definition of the scaffold and transport domains, as described above, and should be applied cautiously if these boundaries are not clear.
(h) *Introduction of the ions and/or substrate*. In the final step, any known ions and/or substrates are introduced into the binding site (**Figure 4**). First, the template substrate position is estimated by superposing the binding site of the known structure on that of the initial model. Then, the model building is repeated using this template substrate, while applying additional distance restraints between substrate atoms and binding site atoms.

The details of the software and parameter choices used are as follows:

*Model building* – Models were built using Modeller v9.13 (Šali and Blundell, 1993). After each of the refinements of the alignment in step f of the protocol, 200 iterations of model building were performed. For steps g and h, 2000 iterations of model building were performed, in order to increase the chances that the sampling creates a model that satisfies all of the input restraints.

*Model assessment* – Each set of models was evaluated using MolPDF and ProQM scores (Ray et al., 2010), as well as Procheck analysis (Laskowski et al., 1993). The MolPDF score describes how well the model satisfies the input restraints, including those created from the template, the alignment, and any additional applied restraints, and is therefore an arbitrary value dependent on those features; the model with the smallest MolPDF score best satisfies all the restraints. The ProQM score measures the degree to which a set of coordinates is consistent with a number of required features, including TM segments predicted using TOPCONS (Bernsel et al., 2009), the distance to the membrane center for residues in α-helical segments predicted with ZPRED (Granseth et al., 2006), secondary structure elements predicted by PSIPRED (Jones, 1999), burial of conserved residues, and exposure of variant residues. The ProQM score is assigned as a 21-residue window-average to each residue, and is then summed to give a total score per model, with values ranging from 0 to 1, where values of 0.7 are typical of membrane protein structures solved by X-ray crystallography. Procheck assesses the degree to which the features of the model are consistent with those of known protein structures in terms of bond distances, angles, dihedrals and overlapping atoms. In this work, Procheck is used to identify the fraction of backbone groups that lie outside the favored regions of the Ramachandran plot.

The final model was selected as that in step h with the lowest MolPDF score, and/or the highest Procheck and global ProQM scores.

*Domain definitions* – The repeats were defined as comprising residues 12-108, 335-416 for repeat unit 1 (RU1) and 150-334 for repeat unit 2 (RU2) in Glt_Ph_, and 119-229 for RU1 and 294-416 for RU2 in VcCNT. The scaffold/oligomerization and transport domains were defined as comprising residues 12-77 plus 129-218 and 78-128 plus 219-416, respectively, for Glt_Ph_, and residues 2-137 plus 241-311 and 138-229 plus 312-416, respectively, in VcCNT.

*Distance restraints* – All distance restraints applied in steps g and h were represented as a Gaussian with a standard deviation of 0.1 Å. Distance restraints between Cα atoms were applied to preserve the internal structure of the scaffold/oligomerization and transport domains, independently, and were assigned according to the input crystal structure for all pairs of Cα atoms < 60 Å apart within each domain.

The distance restraints applied in step h between substrates and binding site atoms were defined as follows:

For Glt_Ph_, restraints were applied between the two Na^+^ ions or any non-hydrogen (“heavy”) atom of the aspartate substrate, and any heavy atom of the protein within 5 Å in the known/template structure, PDB (Berman et al., 2000) identifier, ID: 3KBC (Reyes et al., 2009).

For VcCNT, restraints were applied between the Na^+^ ion and the O atoms in the backbone of N149, V152 and I184, the hydroxyl group of S183, and a crystallographic water molecule. Restraints were also applied between any heavy atoms of the uridine and any heavy-atom of the protein within 3.5 Å in the template structure, PDB ID: 3TIJ (Johnson et al., 2012).

*Other programs* – Structural alignments were obtained using TM-align (Zhang and Skolnick, 2005) and Jalview was used for editing and visualization of alignments (Waterhouse et al., 2009). Secondary structural elements were assigned with DSSP (Edgar, 2010) and conservation patterns were obtained using the ConSurf server with default parameters (Ashkenazy et al., 2010). Molecular figures were generated using PyMol v1.6 (Schrödinger, Inc.). The position of the proteins in the membrane was determined for the X-ray structures with the OPM server (Lomize et al., 2006), and the orientation of the models was defined after superposition of the scaffold domain onto that of the corresponding X-ray structure.

## Results and Discussion

Crisman et al. (2009) previously applied an *ad hoc* repeat-swap protocol to construct an inward-facing substrate-bound occluded model of Glt_Ph_ (Protein Model Database (Castrignanò et al., 2006), PMDB identifier: PM0075966) based on an available structure of Glt_Ph_ in an outward-facing substrate-bound occluded conformation (Boudker et al., 2007, PDB ID: 2NWL). The predicted inward-facing state captured the major conformational change revealed in a structure published around the same time (Reyes et al., 2009), thereby demonstrating that the conformational change is a result of asymmetry exchange. Here, we propose a more standardized and generalized protocol for repeat-swap homology modeling (see Materials and Methods), and calibrate the method by building an outward-facing occluded model of Glt_Ph_ based on the available inward-facing structure (PDB ID: 3KBC), thereby providing a measure of the expected accuracy of the method for that case by comparison with a known structure in the same state (PDB ID: 1XFH). We then apply the updated protocol to VcCNT, revealing that the mechanism of this secondary transporter is also likely to involve an elevator-like conformational change.

### An Outward-Facing Model of Glt_Ph_ Built Using the Inward-Facing Structure as a Template

As mentioned above, structures of Glt_Ph_ are available in several different conformations, including outward- and inward-open, outward-occluded, intermediate, and apo forms (Yernool et al., 2004; Boudker et al., 2007; Reyes et al., 2009; Boudker and Verdon, 2010). Here, we wished to compare predictions of the major conformational change between outward-occluded and inward-occluded states, with structures of the end points at a similar resolution. Thus, we elected to use the inward-occluded substrate-bound structure of Glt_Ph_ at 3.51 Å resolution (PDB ID: 3KBC, Reyes et al., 2009) as a template to model the outward-occluded substrate-bound state, which could then be compared with a known structure also at 3.50 Å resolution (PDB ID: 1XFH, Yernool et al., 2004). This we did by following the protocol described in the Section “Materials and Methods.”

As mentioned above, a few steps in the repeat-swap homology modeling protocol do not yet have reliable automated procedures. Specifically, we defined the inverted-topology repeats in Glt_Ph_ according to Crisman et al. (2009; **Figure 3A**). We note that this differs slightly from the definition of Reyes et al. (2009), who defined TM3 and TM6 as part of RU1B and RU2B, respectively. We elected to define them this way because the structure superpositions led to more reasonable initial sequence alignments, requiring fewer cycles of refinement. The final refined sequence alignment is shown in **Figure 3D**.

For assigning the domains that move relative to one another (transport and scaffold domains) we used the definition of Reyes et al. (2009; **Figure 1**, see Materials and Methods). In an important update to the protocol, restraints were imposed between all pairs of Cα atoms within each of these domains separately during model building. The final model contains aspartate and two sodium ions (**Figure 4A**).

### Accuracy of the Repeat-Swapped Model of Glt_Ph_

**FIGURE 5:**
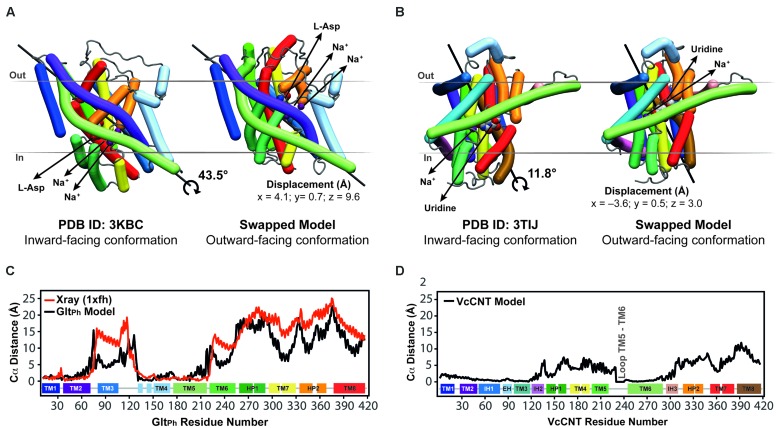
**Crystal structures in an inward-facing conformation compared with the final repeat-swapped models of an outward-facing conformation. (A,B)** The X-ray crystal structures of a protomer of **(A)** Glt_Ph_ and **(B)** VcCNT in an inward-facing conformation (*left*) are compared with the repeat-swapped models of an extracellular-facing conformation (*right*). Structures are viewed along the plane of the membrane, with the extracellular side at the top. The Bendix plugin to VMD was used to represent the helices (Dahl et al., 2012). To quantify the conformational change of the two transporters, the rotation axis, rotation angle, and the displacement of the helices were evaluated. The black line shows the rotation axis, which lies approximately perpendicular to the symmetry axis in each transporter (see **Figure 3B**). The displacement values suggest an elevator-like movement of the transport domain containing the substrates (*spheres*), and this displacement is associated with a rotation of 43.5 and 11.8°, for Glt_Ph_ and VcCNT, respectively. For reference, the conformational change in the transport domain between two X-ray structures of Glt_Ph_ (PDB IDs 3KBC and 1XFH) involves a rotation of 39.6° and a displacement by x = 3.9, y = 2.8, z = 16.4 Å. **(C,D)** Displacement per residue determined as the distance between Cα atoms in the crystal structure and the model after fitting using the scaffold domains (*black*), for Glt_Ph_ **(C)** and VcCNT **(D)**. In the case of Glt_Ph_, the conformational change between known inward-facing and outward-facing substrate-bound structures is shown (*red*).

The model of the outward-occluded state of Glt_Ph_ (**Figure 5A**) was compared with the inward-occluded (template) X-ray crystal structure by structurally aligning the scaffold/oligomerization domains. This comparison revealed a conformational change consisting of a ~44° rotation of the transport domain about an axis running approximately perpendicular to the symmetry axis, and a ~10 Å translocation (normal to the membrane), relative to the scaffold/oligomerization domain. As a result, the substrate binding site is exposed to the opposite side of the membrane. This domain movement translates to positional changes of between 5 and 23 Å for the individual Cα atoms in the transport domain (**Figure 5C**, black line). The helical hairpins HP1 and HP2, which are at the interface with the scaffold domain, undergo the largest movements, whereas the helices facing the lipids (TM3 and TM6) undergo the smallest changes.

These predicted changes recapitulate those observed in a comparison of the template inward-occluded structure (PDB ID: 3KBC), with an outward-occluded substrate-bound structure of similar resolution (PDB ID: 1XFH), which reveals a rotation of ~40° and a translation perpendicular to the membrane of ~16 Å, corresponding to positional changes of between 10 and 25 Å per Cα atom (**Figure 5C**, red line). Thus, the degree of rotation and direction of translocation are very similar. Moreover, this comparison allows a quantitative analysis of the accuracy of the repeat-swap method. Two factors determine the accuracy of the model: first, the degree to which the two repeats are structurally similar, which in turn is related to their sequence similarity; and second, the degree of symmetry that relates the two states.

With respect to the first factor, the similarity between the structures of the repeats is related to the expected accuracy of a typical homology model, which decreases as a function of decreasing sequence identity (Forrest et al., 2006). In Glt_Ph_, the sequence identities between repeats are only ~9–14% (**Figure 3**), leading to an expected accuracy of ~1.5–4 Å in the Cα positions in the TM segments (Forrest et al., 2006). However, with the new protocol we have also captured information about the known structural features of each domain, by incorporating restraints within the oligomerization/scaffold and transport domains separately, based on intra-domain distances in the known structure (see Materials and Methods). During the modeling, these intra-domain distance restraints will be satisfied as long as they do not lead to significant violations of the other restraints obtained from the use of the other repeat as a template (for which there are a larger number of distance restraints). Applying these new intra-domain restraints in the case of Glt_Ph_ leads to a significant improvement in the accuracy of the individual domains. Specifically, after applying these restraints the root mean squared deviation (RMSD) between the model and the crystal structure of the outward-facing conformation decreases from 4.8 Å and 6.7 Å, to 2.5 Å and 2.9 Å over all Cα atoms in the scaffold/oligomerization and transport domains, respectively. This improvement occurs without altering the overall elevator-like movement; before incorporating the intradomain restraints, the rotation and translocation were ~45° and ~11 Å, respectively (cf. ~44° and ~10 Å for the final model described above).

The above results show that the error in the new model is only slightly higher than the difference between the two known structures in different conformations (PDB IDs: 1XFH and 3KBC), which is 1.2 and 3.4 Å for the scaffold/oligomerization and transport domains, respectively. Using a length-independent measure for the same comparison, the template modeling score (TM-score) increases from 0.62 and 0.64 to 0.83 and 0.86, for the models built without and with intra-domain restraints, respectively. (A TM-score of 1.0 indicates that the Cα atoms have identical positions.) For comparison, the TM-scores when comparing the inward- and outward-facing crystal structures domains are 0.91 and 0.73, for the scaffold/oligomerization and transport domains, respectively. The remarkable accuracy of this modeling procedure is achieved in spite of the extremely low (<15%) sequence identity, and even while the overall conformational change is captured (**Figure 5**).

As mentioned, the second factor determining repeat-swap model accuracy is the particular conformational state of the template. Thus, if the input structure is semi-occluded, then the output model will also be semi-occluded. In the case of the swapped model of Glt_Ph_, the rotation of the transport domain is very similar to that observed in comparing the two structures, but the translocation of that domain implied by the model is slightly (~7 Å) smaller (**Figure 5C**, compare red and black lines) demonstrating that the modeled conformational change is less extreme than expected based on the crystal structures. In other words, the (template) inward-facing structure is less “inward” than the known outward-facing structure is “outward,” resulting in a model that is also less “outward.” Thus, the major value of the repeat-swap modeling is to identify the elements involved in the conformational change, and the directionality of their movement, which are clearly both well captured by this approach.

### A Model of the Outward-Facing Conformation of VcCNT

In Johnson et al. (2012), a structure of a CNT from *V. cholerae* (VcCNT) was reported at 2.4 Å resolution, with a substrate binding site that is exposed toward the presumed cytoplasm. VcCNT is related to transporters of the human solute carrier SLC28 family. To date, all other structures reported for this transporter fold reflect the same state of the transporter (Johnson et al., 2014) and therefore the mechanism of conformational change is unknown. It has been proposed, based on visual analysis of the structure and binding site accessibility data, that CNTs undergo a similar elevator-like movement (Johnson et al., 2012), with two helical hairpins moving above and below TM helix 6, which serves as a hydrophobic barrier. In particular, the overall “design principle” of the CNTs appears to be similar to that of Glt_Ph_, with a trimeric quaternary structure, helical hairpins, and clear transport- and scaffold-like boundaries (Johnson et al., 2012, **Figure 1**). However, at the level of individual helices, a structural comparison revealed few commonalities (Johnson et al., 2012). Nevertheless, the VcCNT fold clearly contains inverted-topology repeats, which together assemble into the transport domain (**Figure 3A,B**). Internal symmetry within the scaffold/oligomerization domain of VcCNT is less clear, except for the interfacial helices IH2 and IH3, and therefore, if additional internal symmetry ever existed, it appears to have been lost during evolution, perhaps because trimerization provides sufficient scaffolding without compromising the transport mechanism. As in Glt_Ph_, the scaffold domain of VcCNT also contains within it the helices involved in forming the physiologically relevant trimer interface (**Figure 1B**).

To pursue the hypothesis that VcCNT undergoes an elevator movement, we built a repeat-swapped structural model using the inward-facing structure as a template, and using the definition of repeats shown in **Figure 3A,C**, by applying our updated protocol (**Figure 2**, see Materials and Methods). The final alignment after initial model-building and refinement (**Figures 4C,D**) is shown in **Figure 3D**.

We defined repeat units 1 and 2 as comprising the transport domain, with the exception of interfacial helices IH2 and IH3, which were assigned to the scaffold domain, together with the peripheral trimerization helices (**Figures 1A** and **3A–C**). Along with restraints to position a uridine substrate and a sodium ion in binding sites formed by the same residues as in the template, we also imposed restraints within each domain to build a refined model that matches as many aspects of the known structure as possible, while capturing the same overall conformational change. The final model of VcCNT (**Figures 5B,D**) is freely available from the PMDB (https://bioinformatics.cineca.it/PMDB/) with the identifier PM0080188, and the model generated without restraints is available upon request.

**FIGURE 6:**
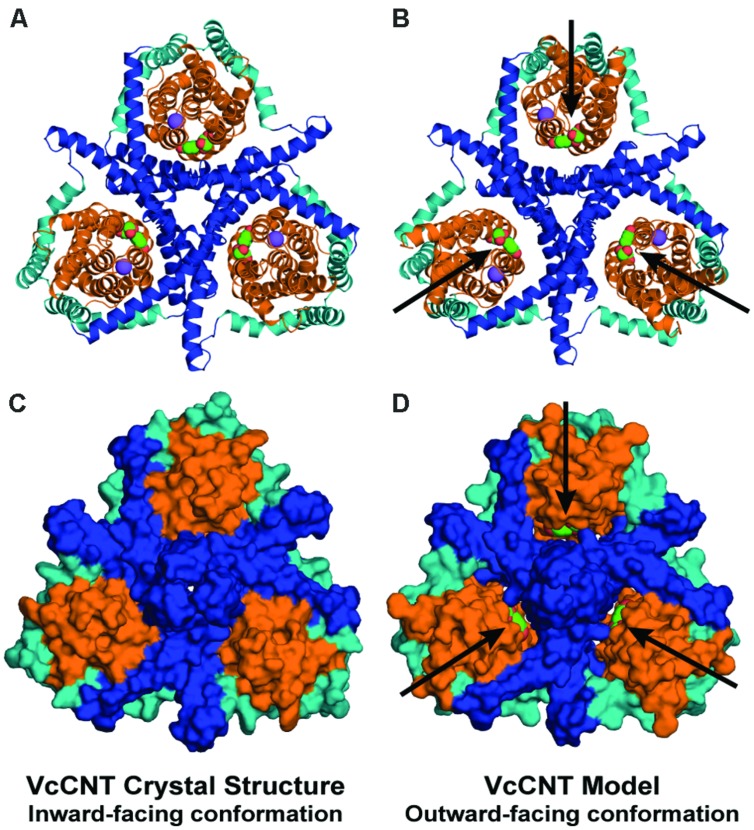
**The repeat-swapped model of VcCNT contains an extracellular substrate pathway that is not present in the template structure.** The trimeric VcCNT X-ray structure **(A,C)** and model **(B,D)** are viewed from the extracellular side, colored according to **Figure 1** and rendered either as cartoon helices **(A,B)** or using the van der Waals surface **(C,D)**. The substrate (*spheres, arrows*) is visible from the extracellular side in the model **(D)**, but not in the inward-facing crystal structure **(C)**.

The repeat-swapped model of VcCNT adopts a conformation in which the substrates are visible via an aqueous pathway connected to the extracellular solution (**Figure 6**). The conformational change predicted by the VcCNT model also involves an elevator-like movement of the hairpin domains, similar to that seen for Glt_Ph_ as suggested by Johnson et al. (2012). These changes arise due to a ~12° rotation of the transport domain around a similar axis to that found in Glt_Ph_, as well as lateral and displacements along the membrane normal of ~3–4 Å (**Figure 5B**). Note that very similar changes are found when the intradomain restraints were not included; specifically, the transport domain rotates by ~15°, and is also translocated by 3–4 Å. Interestingly, the extent of this conformational change is more modest than that of Glt_Ph_, with Cα displacements of between 4 and 10 Å, after superposition on the scaffold helices (**Figure 5D**).

It is entirely possible that VcCNT undergoes more extreme movements than can be predicted based on the available structural data. Nevertheless, as noted above, the directionality and composition of the moving domains predicted by the repeat-swap model are likely to be correct, based on our observations using Glt_Ph_. Therefore, this model of the outward-facing state of VcCNT should provide a useful starting point for experimental studies of the conformational change required for transport in CNTs, by illustrating which elements become buried or exposed, and which elements move relative to one another.

## Conclusion

Repeat-swap modeling has proven to be helpful for analyzing the conformational changes in various homologs of neuronal transporters, including Na^+^/Cl^-^-dependent transporters of the NSS family (Forrest et al., 2008), EAATs (Crisman et al., 2009), and vesicular monoamine transporters (Yaffe et al., 2013). Although the models generated may not be high-resolution enough for drug design, the identification of alternate conformations and conformational mechanisms is essential for the development of treatments against such transporters, as it provides insight into changes in the transporter structure that may affect inhibitor binding. For example, inhibitors of serotonin uptake such as ibogaine and cocaine bind to different conformations of SERT (Tavoulari et al., 2009), and similar conclusions have been evoked for reserpine and tetrabenazine binding to the vesicular monoamine transporter VMAT2 (Schuldiner et al., 1995). Therefore, molecular models based on repeat-swap modeling can be extremely useful to help design further experiments such as cross-linked constructs for crystallography, whose structures can then be used for drug design.

The current study provides an updated and more systematic protocol for repeat-swap modeling of secondary transporters with inverted-repeats. Our analysis of the Glt_Ph_ model reveals that the model accuracy can be dramatically improved by inclusion of restraints extracted from the input structure. Those restraints can be readily defined if there is a distinct boundary between the mobile and static segments of the transporter. In the cases of Glt_Ph_ and VcCNT this boundary became clear after producing an initial repeat-swapped model of each protein, and comparing that model with the input crystal structure by superposition of the helices forming the oligomerization interface. With the benefit of this insight, this boundary becomes relatively clear merely from analysis of the architecture viewed from above the membrane (**Figure 1B**).

The inward-facing crystal structure of VcCNT and the swapped model in an outward-facing conformation that we present here provide a molecular-level description of the conformational changes in CNTs. We expect that this insight will prove to be important for understanding the mechanism of TM transport of nucleosides and of nucleoside-derived drugs, such as anticancer drugs like gemcitabine. More broadly, our model strongly suggests that the remarkable elevator-like movements identified in the glutamate transporter family are not unique, and that transporters in the SLC28 CNT family also undergo elevator-like movements during their alternating access mechanism. This raises the possibility that other transporter families may use a similar elevator-like mechanism. For example, the sodium-coupled dicarboxylate transporter VcINDY (Mancusso et al., 2012), which is a homolog of the neuronal sodium-coupled citrate transporter NaCT, contains inverted-topology repeats with reentrant helical hairpins similar to those observed in Glt_Ph_ and VcCNT. It will be of interest to examine which other neuronal transporters use elevator mechanisms for TM transport of their substrates.

## Conflict of Interest Statement

The authors declare that the research was conducted in the absence of any commercial or financial relationships that could be construed as a potential conflict of interest.

## Abbreviations

CNT: concentrative nucleoside transporter
EAAT: excitatory amino acid transporters
NSSs: neurotransmitter sodium symporters
SERT: serotonin transporter
TM: transmembrane
VcCNT: *Vibrio cholerae* concentrative nucleoside transporter.
